# Endoscopic Middle Ear and Mastoid Surgery for Cholesteatoma

**Published:** 2013

**Authors:** Hamed Sajjadi

**Affiliations:** 1 *Otology/Neurotology- Skull Base Surgery, Stanford University School of Medicine, Stanford, US.*

**Keywords:** Cholesteatoma, Ear surgery, Endoscopic

## Abstract

**Introduction:**

To reduce incidence of residual cholesteatoma following ear surgery; and to reduce the need for second look “open” mastoidectomy using endoscopic mastoidotomy. Ten-year retrospective chart review of 249 primary cholesteatoma cases (1994-2004) with a minimum follow-up of two years. The first objective was to evaluate the effectiveness of otoendoscopy in reducing the incidence of “cholesteatoma remnant” at the time of primary surgery. The second investigation was to evaluate the effectiveness of otoendoscopy in reducing the need to open the mastoid cavities during “second look operations”.

**Materials and Methods:**

Endoscopes were used on all cases as an adjunct to standard microscopic methods. Once all visible cholesteatoma was removed with standard microscopic techniques, endoscopes were utilized in order to identify any “remnants” of cholesteatoma. Endoscopes were also employed during revision second look cases in order to allow the evaluation of intact canal wall mastoid cavities without an open Mastoidectomy approach.

**Results:**

Endoscopy at time of primary operations revealed a 22% incidence of hidden cholesteatoma “remnants” despite apparent total microscopic eradication in closed cavity cases, and 10% in open cavity patients. Endoscopic removal of cholesteatoma remnants reduced the long term cholesteatoma “residual” to 9.7% in closed cavity patients. Furthermore, endoscopic surgery significantly reduced the need to open the mastoids during second look operations.

**Conclusion:**

Otoendoscopy is a very effective adjunctive method in ear surgery. It allows significant reduction in cholesteatoma residual rate in both closed cavity and open cavity cases. Furthermore, the great majority of second look mastoids can be evaluated endoscopically and thus avoid an open revision Mastoidectomy.

## Introduction

Long-term eradication of cholesteatoma remains a noteworthy challenge for otologists. Cholesteatoma has proven to be a formidable enemy. Invasive cholesteatoma can erode bone, destroy ossicles, and invade the inner ear or the facial canal causing facial paralysis, vertigo, and total sensorineural hearing loss. Surgical dealing of cholesteatoma centers on two different approaches. The closed-cavity technique preserves the normal anatomy of the ear canal and allows normal location for the eardrum, thus improving the chances of reconstruction of hearing. The open-cavity technique has numerous variations including the old standard radical mastoidectomy, rarely done nowadays, as well as variations of modified radical mastoidectomy including the intact-bridge tympanomastoidectomy as popularized by Paparella.1

The disadvantage of the closed-cavity technique is the inability to monitor residual cholesteatoma in the post-operative phases through standard office examination. On the other hand, an open-cavity technique allows for relatively straightforward post-operative monitoring of any cholesteatoma residual in the area of the attic. Another major disadvantage to the closed-cavity technique is the almost obligatory need for second-look surgery six to 10 months following the initial surgery to identify any residual cholesteatoma. This is often a difficult task for patients, especially if the hearing is relatively good and the patient has no clinical signs or symptoms; since many patients are reluctant to undergo a major second-look operation. Studies have repeatedly shown, however, that the incidence of residual cholesteatoma following closed-cavity procedures varies from 10% to 43% (2).

Due to the significantly high probability of residual cholesteatoma following closed-cavity procedures, it is of paramount importance to follow patients closely and perform the second-look operation. The endoscopic approach to the middle ear and mastoid has been utilized by several investigators in recent years in an attempt to reduce the morbidity of a second-look operation (3). 

In the late 1970s in Europe, endoscopy became available for some office-evaluations of ear-patients and a rare evaluation in the operating rooms for diagnostic purposes only (4). Thomassin and colleagues in 1987 in France devised the first endoscopically guided otosurgery in the prevention of residual cholesteatoma.^5^ Thomassin compared 36 cases of surgery for cholestea- toma performed from 1985 to 1991 to 44 cases of cholesteatoma take delivery of surgery without endoscopy from 1979 to 1985. In the first series, without the endoscopes, residual cholesteatoma was detected in 47% of cases, compared with 6% in the second series where the combination of endoscopes and microscopic approaches had been used. This landmark article showed a considerable reduction in residual cholesteatoma attributed to the advent of endoscopic evaluation of blind spots encountered during the primary surgery.

In 1993, McKennan reviewed 12 second-look tympanomastoidectomies following cholesteatoma and performed endoscopic mastoidoscopy through a small post-auricular incision using Richard telescopes.^6^ These 12 patients underwent conventional standard microscopic tympanomasto- idectomy and facial recess surgery to eradicate cholesteatoma. Endoscopes were used in the second-look operation, and two of the 12 cases were noted to have cholesteatoma which was treated endoscopically. McKennan concluded that this endoscopic mastoidoscopy through a small post-auricular incision reduced surgical morbidity and allowed for faster recovery and healing with reduced pain and discomfort for the patients. Bottrill and Poe in 1995 reported endoscopic assisted ear-surgery in nine cases (7). The authors considered the endoscopes were beneficial and reduced the morbidity as well as increased the accuracy of surgical resection. Youssef and Poe in 1997 reported a much larger study of 19 cases that were managed endoscopically out of 25 patients with second-look operations. Their view was that the 13 cases that were explored endoscopically should be opened up, and a standard mastoidectomy was performed in these cases. They reported 23% residual cholesteatoma detected on endoscopic evaluation. Following the endoscope proved that there was no cholesteatoma and the mastoids were opened, there were no surprises and no false-negatives (8). 

Tarabichi in 1997 discussed endoscopic management of acquired cholesteatoma (9). In this study, 25 cases were managed endoscopically and a two-year follow-up was obtained in 14 of those patients. Tarabichi’s did a completely endoscopic tympanoplasty and mastoidectomy on the primary case for cholesteatoma. Eleven of the 25 patients presented with extensive cholesteatomatous disease within the mastoid cavity as well as the middle ear. Transcanal atticotomy was then performed, and endoscopic resection of the cholesteatoma was commenced. The author did not report any significant complications associated with the total of 36 endoscopic procedures that were performed. At the two-year follow-up, four out of the six patients followed at that time were free of disease. The author’s recommendation of completely endoscopic surgery replacing microscopic surgery had not been widely accepted.

Haberkamp et al. in 1999 reported a limited experience with endoscopic second-looks following primary conventional procedures using tympanomastoidectomy (10). The incidence of residual cholesteatoma was 20% in the endoscopic cases and 50% in the traditional. No false negatives were detected after microscopic evaluation was performed following endoscopic surgery. The authors concluded that “endoscopic mastoidoscopy offered an alternative to the traditional revisional mastoidectomy and second-look surgery after intact-canal-wall mastoid- ectomy.”

Rosenberg and Silverstein in 1995 studied the use of endoscopes in pediatric surgery for chronic ear problems (11). Ten patients underwent an endoscopic evaluation, as well as a standard open-mastoidectomy procedure during the same operation, to investigate the accuracy of the endoscopic method. Results revealed that the endoscopic findings correlated exactly with the open mastoidectomy techniques in all cases. The conclusion of the study was that “open second-look mastoidectomy might be avoided if minimal or no cholesteatoma is found during the endoscopic exploration at the second look.”

Yung in 1994 reported a relatively large study of 92 cases of cholesteatoma employing 64 intact-canal wall procedures and 28 canal-wall-down procedures with the endoscopic method (12).The author posited that the use of angled rigid endoscopes in conjunction with the operating microscopes would facilitate removal of cholesteatoma during primary mastoid operations. This article involved mainly canal-wall-down surgery. Sixty-four patients out of the 92 had a small cavity mastoidectomy; 18 had open-cavity mastoidectomy with reconstruction of the canal wall; and ten other patients had open-cavity mastoidectomies with primary obliteration. No intact-canal-wall surgery was performed. Endoscopes were utilized following microscopic resection of cholesteatoma. A total of three cases of cholesteatoma were detected, one in each category of his patients.

In 2001, Yung reported a larger series of endoscopically managed cases of cholesteatoma (13). The objective of that study was to determine whether residual cholesteatoma had been eliminated by the use of endoscopy in the middle ear. Yung reported 231 primary operations for cholesteatoma performed from 1988 to 1999. These operations included closed-cavity mastoidectomy in 53 cases and small-cavity mastoidectomy in 115 patients. Open mastoidectomy with primary reconstruction of the canal wall was also accomplished in 44 patients, and mastoid obliteration in 19. The mean follow-up here was 6.5 years. Yung reported the incidence of residual cholesteatoma at 9.4%, which was very similar to results for the open-cavity mastoidectomy incidence of 8.7%. This study revealed that residual cholesteatoma, although not eliminated by the advent of Endoscopy, has been reduced significantly to levels similar to those for closed-cavity techniques.

## Materials and Methods

This study was a retrospective chart review to investigate “residual” cholesteatoma and not “recurrent” one. The institutional review board was not presented since standard otologic methods were used to treat patients, and endoscopy was used as an adjunctive technique. Cholesteatoma “residual” is defined as that cholesteatoma detected during the second look operation at the same site as the primary surgery months earlier. “Recurrent” cholesteatoma is “new” disease that is found in a new attic defect, eardrum retraction pocket or around Ossicles or middle ear prosthesis. 

Recurrent cholesteatoma was excluded in this study. Post-operatively “recurrent” cholesteatoma is a very complicated, multifactorial phenomenon and was not addressed in this research. The current investigation was to appraise why and how cholesteatoma is left behind during the initial operation thus leading to “residual” cholesteatoma later on. 

Wide-angle rigid 2.7 mm and 4 mm diameter, zero, 30, and 70 degree endoscopes coupled with a 3-chip video camera and high definition monitor, were used intra-operatively. Furthermore, zero and 30-degree rigid ear-telescopes were also used on all patients pre-operatively in the office setting. These endoscopes are widely available from a variety of vendors. 

From 1994 to 2004, all surgeries for cholesteatoma were performed using both microscopic and endoscopic methods without any exceptions. All patients undergoing ear surgery for cholesteatoma would undergo a traditional microscopic resection as indicated. Once the microscopic resection was completed and there was no visible cholesteatoma under the microscope, then the endoscopes utilized to evaluate all areas.

Rigid endoscopes were used to assess the entire middle ear space including the sinus tympani, the mesotympanum, hypotym- panum, orifice of the eustachian tube, the attic, and around the ossicles. Once endoscopy was completed in the middle ear, if the mastoid had been opened during the initial surgery then endoscopy was also utilized in the mastoid to look at the attic and the area of the cog to make sure there was no residual cholesteatoma. 

A total of 249 primary surgeries for cholesteatoma over a ten-year period were reviewed. Twenty-nine patients were lost to follow-up. Two hundred twenty ears underwent “second look” surgery within 10-18 months following the primary operation. This allowed review of 469 cases of cholesteatoma managed with both standard and endoscopic methods. 

There were 224 patients with 249 primary ears, with 25 bilateral cases. One hundred sixteen males and 108 female with an age range of 4 years to 82 years old were treated. Type and location of cholesteatoma included 144 attic cholesteatoma, 67 posterior inferior retraction pockets with keratin debris, 22 congenital cholesteatoma and 16 cases of anterior quadrant cholesteatoma had been documented. 

*Distribution of cases: *Two groups of cases were defined: closed cavity and open cavity. Closed cavity cases were defined as all patients who had intact canal wall surgical resection of cholesteatoma. This included any combination of atticotomy with repair, middle ear cholesteatoma resection, with or without intact canal wall mastoidectomy. Open cavity patients included cases when the posterior ear canal wall was removed, with or without mastoid obliteration.

All patients were strictly informed pre-operatively that a second-look surgery was an essential part of their treatment protocol. All patients had consented to undergo the second-look surgery before the primary operation was performed. A primary operation using a closed cavity technique was done in 182 patients. Of the 182 primary closed cavity cases, 15 patients were lost to follow-up and/or refused a second-look procedure. 

A total of 67 patients underwent a variation of open cavity technique. In these canal wall down mastoidectomy cases, 57 patients had the intact-bridge mastoidectomy, with mastoid obliteration using bone paté as described by Sajjadi (14). Ten patients underwent standard canal-wall-down surgery with mastoid obliteration. Of these 67 canal wall down patients, 14 were lost to follow-up. No radical mastoidectomy was done. All middle ear perforations were grafted and no “exteriorized middle ear case” was performed (Table 1).

**Table 1:** Surgical Case Distribution (total 459 operations

**Typial Sarger **	**Namber**
1. Primary closed cavity	182
Second look closed cavity	167
Closed cavity lost to follow up	15
2. Primary open cavity	67
Second look open cavity	53
Open cavity lost to follow up	14
Total	249

Attic cholesteatoma had been observed in 144 cases. Of these patients, 45 cases underwent attico-antrotomy (inside-out) resection with cartilage repair. Ninety nine attic cholesteatoma cases underwent mastoidectomy. Of these mastoidectomy cases, 64 cases underwent canal wall down technique and 35 patients had intact canal wall method. There were 67 cases of cholesteatoma in posterior inferior ear drum retraction pocket adherent onto promontory and stapes supra-structure / remnant. Among these patients 61 cases were managed with middle ear resection and cartilage repair without mastoidectomy. The remaining 6 cases underwent mastoidectomy, with 3 canal wall down cases and 3 intact canal wall cases.

There were 22 cases of congenital cholesteatoma. Fifteen of these cases were restricted to the middle ear and were managed with trans-tympanic resection and reconstruction without mastoidectomy. The remaining 7 cases underwent intact canal wall mastoidectomy. The last group included 16 patients with anterior quadrant retraction pockets with cholesteatoma; all these were managed with trans-tympanic middle ear resection and repaired without mastoidectomy. A total of 45 intact wall mastoidectomies and 67 canal wall down mastoidectomies were ensured at the time of primary operation. The great majority of patients, 137 cases, with cholesteatoma had primary resection and repair without mastoidectomy (Table 2).

**Table 2:** Location of Cholesteatoma

**Typial Sarger**	**Namber**
1. Attic cholesteatoma	144
A. Attico-antromy	45
B. Mastoidectomy	99
ICW	35
CWD	64
2. Posterior TM collapse	67
A. Middle ear resection	61
B. Mastoidectomy	6
ICW	3
CWD	3
3. Congenital cholesteatoma	22
A. Middle ear resection	15
B. Mastoidectomy (ICW)	7
4. Anterior TM cholesteatoma	16
All via middle ear resection	
Total	249

Intra operative Technique: All patients underwent standard otologic surgery using the microscopic techniques. Once complete resection of cholesteatoma was accomp- lished, otologic endoscopes were used to check for any cholesteatoma remnants. Closed cavity technique was performed in 182 out of the 249 cases. Cholesteatoma was removed from the attic through combination anterior and posterior atticotomy. No “routine” facial recess approach was performed; however,cholestea- toma detected in the facial recess air cells was resected and facial recess opened up as indicated.

Once there was no more cholesteatoma detected through the microscope, endoscopic evaluation was performed. Endoscopes were attached to a video monitor that was held in the surgeon’s left hand. Endoscope-holders were not used. High definition monitors with high-resolution, 3-chip video cameras were used in most cases.

## Results

For the 182 cases receiving primary closed cavity technique, once the microscopic cholesteatoma was resected and the surgeon was confident there was no visible cholesteatoma, endoscopy revealed a 22% incidence of cholesteatoma residual at the time of the primary operation (40 patients). The distribution of the residual cholesteatoma was 55% in the sinus tympani (22 patients), 30% in the attic (12 patients), and 15% in the cog areas (6 patients) (Table 3) .

**Table 3:** Cholesteatoma Remnants Found on Endoscopy

**Typial Sarger**	**Namber**
1. Close cavity cases (n=182)	40 (22%)
Location:	
Sinus Tympani	22 (55%)
Attic	12 (30%
Cog area	6 (15%)
2. Open cavity cases (n=67)	7 (10%)
All located in sinus tympani	
Total	47(32%)

In the closed cavity group of 67 cases, 7 patients (10%) were noted to have cholesteatoma remnants following attempted “complete” microscopic resection. All these remnants were in the sinus tympani area in canal wall down cases. It is safe to assume that the 40 positive cholesteatoma remnant cases (22% incidence) in the closed cavity group and the 7 cases in the open cavity group, if untreated, would have eventually lead to “residual” cholesteatoma in the 

future. This is a relatively typical incidence of residual cholesteatoma as reported in current literature following use of microscopic techniques (15). 

Of the 167 Closed cavity cases that underwent a second-look procedure, 45 had intact canal wall mastoidectomy cavities to be looked at. Of these intact canal wall mastoids, 5 mastoids were unable to be scoped due to scar-tissue and difficulty gaining access, and these underwent open post-auricular mastoidectomy, with an incidence of 10% inability to evaluate the mastoid endos- copically. These scarred mastoids proved to be free of disease once opened up. 

The remaining 40 mastoids were scoped successfully. Four of these mastoid endos- copy cases indicated residual cholesteatoma and were subsequently opened up. All four cases had cholesteatoma deep in the posterior attic area as seen on endoscopy (Table 4).

**Table 4:** Mastoid Disease Status on Second Look Surgery

**Typial Sarger**		**Namber**
1. Intact Canal Wall Mastoids	45	
A. Scoped successfully	40	
B. Unable to scope	5	
C. Positive Mastoids	4	
D. Residual rate 4/45	9	
2. Canal Wall Down Mastoids	53	
A. All opened traditionally		
B. Positive mastoids	0	
C. Residual rate	0	
Total	98	

The total rate of residual cholesteatoma on second looks after primary closed cavity techniques was 9.7% (16 patients). Eleven of these patients had small cholesteatoma pearls in the attic or sinus tympani, which were eliminated using the endoscopic method. Five cases had more extensive cholesteatoma requiring an open approach to the mastoid through combined microscopic and endoscopic techniques. 

Of the 67 canal-wall-down mastoid- ectomies, 14 patients were lost to follow-up, leaving 53 patients for second-look procedures. Three of the 53 (an incidence of 5%) had small residual cholesteatoma pearls in the sinus tympani (Table 5).

**Table 5:** Cholesteatoma Residual Rate following the Endoscopy

**Typial Sarger**	**Namber**
1. Closed cavity cases (n= 167)	16 (9.7%)
Small pearls	10
Extensive disease	6
2. Open cavity Cases (n=53)	3 (5%)
All pearls in sinus tympani	
Total	19(14.7%)

*Third Look Surgery:* Planned “third look surgery” was offered to all closed cavity patients who had positive disease at the time of their second operation. In the closed cavity group of 16 positive second looks, 6 patients refused a third operation and were lost to follow-up. Ten patients underwent a third operation about a year following the second surgery. Only one patient had minimal “residual” disease and subsequently underwent endoscopic resection of a small pearl and is free of disease four years post-operatively. No third look surgery was done on the three canal wall down patients who had positive minimal disease at their second operation. They are clinically free of disease with a minimum three year follow-up. 

This data strongly suggests that a negative second look surgery for “residual” cholesteatoma is most likely a very accurate assessment and no further surgery is required. This study excluded cases of post-operatively acquired “recurrent” cholesteatoma in new attic defects or newly acquired eardrum retraction pockets. 

## Discussion

Intra-operative endoscopic evaluation of patients with cholesteatoma has clearly demonstrated a significant reduction in “immediate remnants” of cholesteatoma at the time of the primary operation. As this study and other studies have indicated,^16^ residual cholesteatoma is still not totally eliminated. However endoscopic resection of cholesteatoma following detailed microscopic surgery has reduced the incidence of residual cholesteatoma detected on second-look surgery to a very low rate of 9.7% in closed cavity cases. 

Canal wall down surgery has a slightly lower incidence of residual cholesteatoma, at 5%. Sinus tympani remain a hot spot for residual cholesteatoma despite removal of the posterior ear canal wall. Endoscopic mastoidotomy allows for significant reduction in morbidity after second-look procedures by allowing not opening 75% of the mastoid cavities. 

There is a negligible risk of residual disease following a second look operation. Consequently, no third look surgery is currently offered to patients with negative second looks.

Middle ear and mastoid endoscopy is a safe and highly effective adjunctive technique to the standard microsurgical dissection. Endoscopic ear surgery requires extra training and effort. Surgeons need to develop the ability to operate one handed, and to differentiate between granulation tissue, tympanosclerotic plaques and true cholesteatoma as seen on endoscopes. 

Proper use of endoscopes, video cameras, and endoscopic instrumentations are essential in achieving successful outcomes. Furthermore, surgeons interested in performing endoscopic ear surgery need to make ear endoscopy a routine part of all otologic cases in order to increase efficiency and familiarization of the entire surgical team with this approach. The learning curve to master endoscopic ear surgery is rather steep. Surgeons are encouraged to take hands-on dissection courses and start slow and gradually increase their reliance on endoscopes. All the basic principles of otologic surgery remain valid and must be adhered to while using endoscopes to augment surgical techniques.
